# GABA_B_ receptor attenuation of GABA_A_ currents in neurons of the mammalian central nervous system

**DOI:** 10.14814/phy2.13129

**Published:** 2017-03-29

**Authors:** Wen Shen, Changlong Nan, Peter T Nelson, Harris Ripps, Malcolm M Slaughter

**Affiliations:** ^1^ Department of Biomedical ScienceCharles E. Schmidt College of MedicineFlorida Atlantic UniversityBoca RatonFlorida; ^2^ Division of NeuropathologyDepartment of PathologyUniversity of KentuckyLexingtonKentucky; ^3^ Sanders‐Brown Centre on AgingUniversity of KentuckyLexingtonKentucky; ^4^ Department of Ophthalmology and Visual SciencesUniversity of Illinois College of MedicineChicagoIllinois; ^5^ Whitman InvestigatorMarine Biological LaboratoryWoods HoleMassachusetts; ^6^ Department of Physiology and BiophysicsState University of New York at BuffaloBuffaloNew York

**Keywords:** GABA_A_ receptors, GABA_B_ receptors, human amygdala, mouse retina, *ρ*‐subunit GABA_A_ receptors

## Abstract

Ionotropic receptors are tightly regulated by second messenger systems and are often present along with their metabotropic counterparts on a neuron's plasma membrane. This leads to the hypothesis that the two receptor subtypes can interact, and indeed this has been observed in excitatory glutamate and inhibitory GABA receptors. In both systems the metabotropic pathway augments the ionotropic receptor response. However, we have found that the metabotropic GABA_B_ receptor can suppress the ionotropic GABA_A_ receptor current, in both the in vitro mouse retina and in human amygdala membrane fractions. Expression of amygdala membrane microdomains in *Xenopus* oocytes by microtransplantation produced functional ionotropic and metabotropic GABA receptors. Most GABA_A_ receptors had properties of *α*‐subunit containing receptors, with ~5% having *ρ*‐subunit properties. Only GABA_A_ receptors with *α*‐subunit‐like properties were regulated by GABA_B_ receptors. In mouse retinal ganglion cells, where only *α*‐subunit‐containing GABA_A_ receptors are expressed, GABA_B_ receptors suppressed GABA_A_ receptor currents. This suppression was blocked by GABA_B_ receptor antagonists, G‐protein inhibitors, and GABA_B_ receptor antibodies. Based on the kinetic differences between metabotropic and ionotropic receptors, their interaction would suppress repeated, rapid GABAergic inhibition.

## Introduction

GABA, the major inhibitory transmitter in brain, binds fast‐acting ionotropic GABA_A_Rs that function as Cl^−^ permeable heteropentameric ion channels. Additionally, GABA activates slower, metabotropic GABA_B_ G‐protein‐coupled receptors (GPCRs) that regulate voltage‐gated K^+^ and Ca^2+^ channels (Bowery et al. 1980; Kaupmann et al. 1998). As their activity influences many neural systems and behavioral states, the GABA_B_R is a major target of therapeutic drugs for mental disorders and drugs of abuse (Bettler et al. 2004).

Both retina and amygdala express high levels of ionotropic and metabotropic GABA receptors (Li et al. 1996). Retinal ganglion cells possess both GABA_A_Rs and GABA_B_Rs (Friedman and Redburn 1990; Koulen et al. 1998), and GABAergic neurons are essential for image processing in ganglion cells (Fried et al. 2005). GABAergic transmission is also crucial for the low firing rate and strong inhibitory tone in amygdala and projection neurons that target the thalamus and hypothalamus (Barnard et al. 1998; Lang and Pare 1998; Quirk and Gehlert 2003) and have been linked to emotional behavior, such as anxiety (Lehner et al. 2010). GABA_B_Rs participate in this action by inhibiting glutamatergic cortical input to lateral amygdala, selectively suppressing excitation of principle neurons (Yamada et al. 1999; Pan et al. 2009). The GABA_B_Rs also suppress projection neurons postsynaptically by activating inward rectifying potassium channels (Huttmann et al. 2006).

The *α*‐subunit containing GABA_A_R possesses consensus sequence phosphorylation sites for PKA, PKC, tyrosine, and calmodulin kinases (Song and Messing 2005). Notably, PKC reduces the GABA_A_R current and this is associated with various serine sites on *β*‐ and *γ*‐subunits (Kellenberger et al. 1992; Krishek et al. 1994). An example is serotonin suppression of GABA currents in prefrontal cortex through PKC activation (Feng et al. 2001). PKA can either enhance or suppress GABA_A_R currents, depending on the *β*‐subunit of the receptor (McDonald et al. 1998).

The GABA_B_R reduces adenylate cyclase activity (Dutar and Nicoll 1988; Kamatchi and Ticku 1990; Knight and Bowery 1996) and modulates PKC (Dutar and Nicoll 1988; Taniyama et al. 1992; Kubota et al. 2003). Consequently, the GABA_B_R can act through a variety of second messenger cascades to modulate the GABA_A_R current. In GABA receptors that contain the *δ* subunit, the GABA_B_R can increase the tonic current of the GABA_A_R and promote inhibition, likely acting by suppressing PKA activity (Connelly et al. 2013; Tao et al. 2013). This has been found in dentate gyrus, thalamus, and cerebellum.

A report on the interaction between GABA_A_ and GABA_B_ receptors in bullfrog dorsal root ganglion neurons was described in 1997 (Xi et al. 1997), but to our knowledge there has been no further exploration of this phenomenon. The purpose of this study was to explore GABA receptor interactions in two mammalian tissues: native mouse retinal neurons and in tissue derived from human amygdala. The latter was accomplished by microtransplantation of plasma membrane from human amygdala into *Xenopus* oocytes (Miledi et al. 2006). The in vitro retina preparation demonstrates this crosstalk between GABA_A_ and GABA_B_ receptors in the mammalian nervous system; the oocyte preparation demonstrates the utility of the microtransplantation technique in examining multireceptor activation in an inaccessible part of the human nervous system. In combination, these experiments indicate that GABA_B_Rs may have a widespread and unanticipated net disinhibitory action in the mammalian central nervous system.

## Materials and Methods

### Microtransplantation of membrane fractions

The microtransplantation method of incorporating transmitter receptors from native tissue into *Xenopus* oocytes was employed. This is an alternative approach for studying ion channel and receptor properties (Miledi et al. 2002, 2004, 2006). The method is designed to insert into the oocytes with already assembled receptors and ion channels in their native membrane fraction, bypassing the oocyte's protein processing machinery elicited by foreign RNA transfection.

Human amygdala tissue was obtained from four males and two females autopsied at the University of Kentucky Alzheimer's Disease (AD) Center biobank, under the purview of the University of Kentucky IRB (Schmitt et al. 2012), but who had no AD pathology. The postmortem intervals were all <4 h, and tissues were snap‐frozen at the time of autopsy in liquid nitrogen and then stored at −80°C until use. Membrane fractions were collected following the published protocol (Eusebi et al. 2009). Briefly, a 500‐ to 600‐mg piece of frozen human amygdala tissue was homogenized in a glass tube containing a high glucose solution. The homogenized solution was centrifuged for 15 min at 9400*g* (Eppendorf Centrifugal 5418) in a cold room, and the supernatant was collected and ultracentrifuged at 100,000 g (Beckman Coulter Optima L‐90K) for 2 h at 4^o^C. The pellets (membrane proteins and lipids) were resuspended in a cold glycine buffer solution and stored at −80°C.

Freshly harvested *Xenopus* oocytes were purchased from the Ecocyte Bioscientific US LLC (Austin, TX). The oocytes were injected with 41–82 nL of membrane fraction samples, in which the protein concentrations were calibrated at 0.5–1 mg/mL, using an autonanoliter injector – Nanoject II (Drummond Scientific Company). After 1–2 days, the native membrane proteins embedded in their natural lipid environment readily incorporated into surface membranes of the injected oocytes. A sham control was performed by injection of a glycine buffer solution.

### Electrophysiological recording

The oocytes were placed in the recording chamber and superfused with modified Barth's solution containing (mmol/L): NaCl (115), KCl (2), CaCl_2_ (1.8), N‐2‐hydroxyethylpiperazine‐N’‐2‐ethanesulfonic acid (HEPES, 5), pH 7.4, at room temperature. GABA currents were recorded from individual oocytes using a double‐electrode voltage‐clamp amplifier (GeneClamp 500B, Axon Instruments, Inc.). Microelectrodes were pulled to resistances between 0.7 and 1.5 MΩ when filled with 3.0 mol/L KCl for voltage and current recordings. Data acquisition and analysis were performed using Powerlab‐LabChat V7 (AD Instruments). Where applicable, drug–receptor interaction curves were determined by fitting the experimental data to a Hill equation:

$${\text{I/I}}_{\max}={\text{[C]}}^{n}/\left({\text{[C]}}^{n}+{\left[{\text{EC}}_{50}\right]}^{n}\right)$$

where *I* is the current response to a drug concentration [C], *I*_max_ is the current elicited at a saturating drug concentration, *n* is the Hill coefficient, and EC_50_ (or 1/IC_50_, where the reciprocal replaces EC_50_ in the above equation) is the concentration at which a half‐maximal drug response is obtained. Average peak current was measured and presented as mean ± SEM of 4–18 sets of data from different oocyte batches. Significant differences were determined by unpaired Student's *t*‐test using Microsoft Excel. The receptor agonists and antagonists were prepared in the modified Barth's solution and were superfused by a gravity‐feed perfusion system.

Whole‐cell recording from mouse retinal ganglion cells was performed with an EPC‐10 amplifier and HEKA software (HEKA). Briefly, the retina was isolated from 4‐ to 8‐week‐old mice and flat mounted on filter paper with photoreceptors down. The retinal tissue and filter paper were vertically sectioned in 250–300 *μ*m slices in cold HEPES‐buffered oxygenated MEM solution (Corning). All procedures were performed in accordance with the provisions of the National Institutes of Health Guide for the Care and Use of Laboratory Animals, and approved by the University's Animal Care Committee.

A single slice was moved to a recording chamber and superfused constantly with the MEM solution. Voltage‐clamp recordings were made on ganglion cells in the retinal slices. The recording electrodes were filled with an intracellular solution containing (mmol/L): K‐gluconate 120, MgCl_2_ 1, CaCl_2_ 0.5, HEPES 10, EGTA 5, GTP 1, ATP 5. GABA_A_ and GABA_B_ receptor agonists were briefly puffed on ganglion cells with a DAD‐VM 12 valve manifold superfusion system (ALA Scientific Instruments). All the chemicals were purchased from Sigma or Tocris.

### Immunoantibody labeling

After voltage or current recording, the oocytes were washed twice with the modified Barth's solution and fixed in a 4% paraformaldehyde solution for 45 min at room temperature followed by washing with the Barth's solution. The oocytes were treated in 1% Triton‐X100 contained Barth's solution for 20 min, immersed in the blocking solution containing 5% goat serum for 2 h, then incubated overnight at 4°C in solution containing rabbit monoclonal anti‐GBRII (GABA_B_ receptor II, Abcam, ab75838, 1:1000) and 5% goat serum. After washing with the Triton‐X100 solution the oocytes were incubated with goat‐anti‐rabbit Cy‐3‐conjugated secondary antibody (1:600) for 1 h in darkness. Immunostained oocytes were visualized in a Zeiss LSM 700 confocal microscope system (Munich, Germany).

## Results

### Characterizing ionotropic GABA receptors from the human amygdala

**Figure 1:**
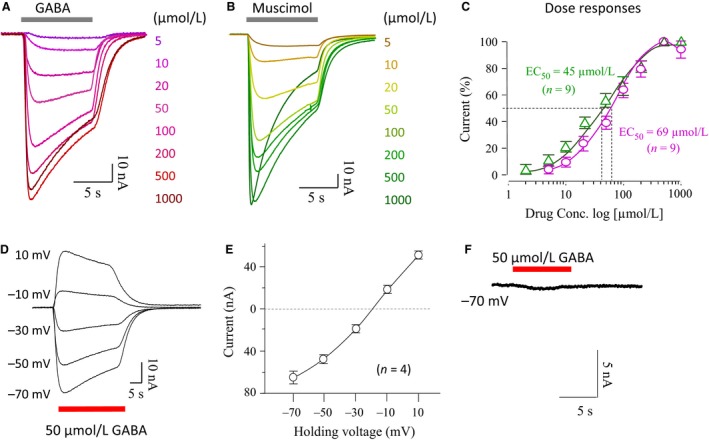
GABA‐ and muscimol‐sensitive responses from the *Xenopus* oocytes transplanted with native human amygdala membrane fraction. Sample voltage‐clamp recordings from *Xenopus* oocytes microtransplanted with human amygdala plasma membrane in response to various concentrations of GABA (A) or muscimol (B). (C) Average dose–response curves of GABA and muscimol. (D) Example of currents evoked by 50 *μ*mol/L GABA at various holding voltages (mV): −70, −50, −30, −10, and 10. (E) Current–voltage relationship of GABA in the transplanted oocytes (*n* = 4). (F) GABA current in the sham‐injected oocytes.

GABA responses in human amygdala have not been reported, so initial experiments were performed to characterize the ionotropic GABA receptor currents. Oocytes were studied 2–3 days after injection of amygdala membrane fractions. As illustrated in Figure 1A, currents were elicited by various concentrations of GABA (5–1000 *μ*mol/L) in transplanted oocytes held at −70 mV. The threshold concentration for GABA was around 5 *μ*mol/L and maximum GABA currents were elicited by 500 *μ*mol/L GABA. The current response to 1000 *μ*mol/L GABA was reduced, probably due to fast desensitization (darkest trace in Fig. 1A). Also, a noticeable GABA current decay appeared when the concentration exceeded 20 *μ*mol/L GABA, indicative of receptor desensitization. A similar pattern of dose‐dependent currents was induced by muscimol, a selective *α*‐subunit GABA_A_R agonist (Fig. 1B). The dose–response curves of GABA and muscimol are plotted in Figure 1C; the calculated mean EC_50_ of GABA and muscimol were 69 *μ*mol/L (*n* = 9) and 45 *μ*mol/L (*n* = 9), respectively. On average, the maximum current amplitudes generated by saturating concentrations (500 *μ*mol/L) of GABA and muscimol were 134 ± 21 nA (*n* = 9) and 189 ± 32 nA (*n* = 5), respectively, representing a statistically significant difference (*P* < 0.01) in the responses to these agonists.

GABA‐elicited currents were recorded at various voltages as designated in Figure 1D. The peak current amplitudes at each voltage are plotted in Figure 1E, showing that the voltage–current relationship of GABA receptors was approximately linear with the reversal potential around −20 mV, corresponding to the Cl^−^ equilibrium potential in *Xenopus* oocytes (Kusano et al. 1982). In a negative control, GABA currents were recorded from oocytes injected with a sham solution, showing that no endogenous GABA response (Fig. 1F).

**Figure 2:**
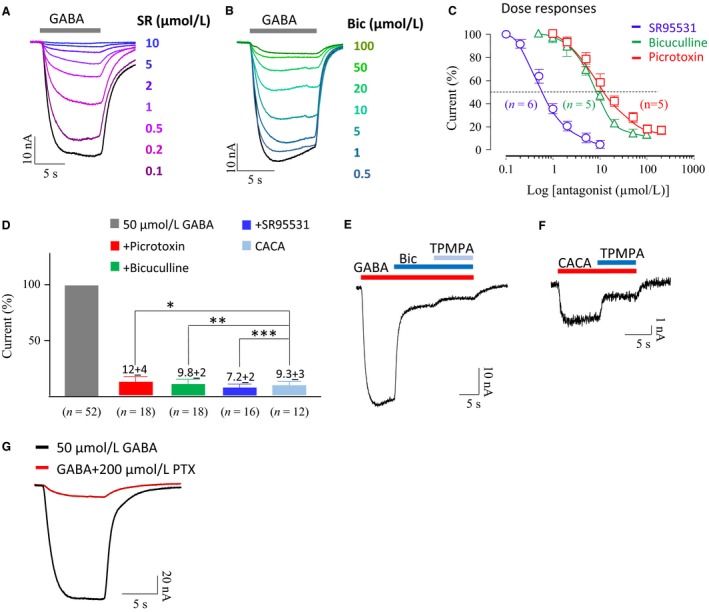
Pharmacology of GABA_A_ receptors from transplanted human amygdala. (A, B) Examples of dose‐dependent inhibition of GABA currents by SR95531 and bicuculline. (C) Dose‐dependent inhibition curves of SR95531, bicuculline, and picrotoxin (PTX) against 50 *μ*mol/L GABA. (D) Histogram shows normalized average GABA currents alone and in the presence of PTX, bicuculline, or SR95531, as well as current produced by CACA (*cis*‐aminocrotonic acid). Statistically there was no difference between the CACA currents and the currents insensitive to picrotoxin, bicuculline, or SR95531 (*, **, *** indicate *P* < 0.05, Student's *t*‐test). (E) A small TPMPA‐sensitive GABA current observed when *α*‐subunit GABA_A_ receptors were blocked by bicuculline. (F) CACA‐sensitive currents were partially blocked by TPMPA. (G) Small GABA current in the presence of picrotoxin.

Ionotropic GABA_A_Rs can be broadly divided into *α*‐subunit containing receptors (the classical GABA_A_Rs) and the more recently discovered *ρ*‐subunit containing GABA_A_Rs (often called GABA_C_Rs). Pharmacological properties of *α*‐containing GABA_A_Rs from human amygdala were studied using SR95531 (gabazine) and bicuculline, selective antagonists. These agents were tested against 50 *μ*mol/L GABA, the approximate EC_50_ concentration (see Fig. 1C). The GABA currents were antagonized by either SR95531 or bicuculline in a dose‐dependent manner (Fig. 2A and B). Picrotoxin, a nonselective blocker of Cl^−^ permeable receptors, was also an effective dose‐dependent inhibitor (data not shown). The antagonist dose–response curves shown in Figure 2C indicate that the IC_50_ of SR95531, bicuculline, and picrotoxin were 0.6 *μ*mol/L (*n* = 6), 8 *μ*mol/L (*n* = 5), and 10 *μ*mol/L (*n* = 5), respectively. The concentrations needed for maximal inhibition (IC_max_) were approximately 10 *μ*mol/L for SR95531 and 100 *μ*mol/L for both bicuculline and picrotoxin (Fig. 2C). However, none of the antagonists fully blocked the GABA‐induced current, generally 10% of the current remained (Fig. 2D). Amygdala is reported to express *ρ*‐subunit GABA_A_Rs (Li et al. 1996; Cunha et al. 2010; Flores‐Gracia et al. 2010). CACA (*cis* 4‐aminocrotonic acid) is a *ρ*‐subunit GABA_A_R agonist (Johnston 1996; Cherubini and Strata 1997). CACA at 100 *μ*mol/L produced a maximal current that was approximately 9% of the EC_50_ current produced by 50 *μ*mol/L GABA, indicating it contributes about 5% of the total GABA current (Fig 2D).

TPMPA (1,2,5,6‐tetrahydropyridin‐4‐yl methylphosphinic acid) is a *ρ*‐subunit GABA_A_R antagonist. In experiments in which the *α*‐subunit GABA_A_R current was blocked by 200 *μ*mol/L bicuculline, then 100 *μ*mol/L TPMPA, could partially block the remaining current produced by 50 *μ*mol/L GABA (Fig. 2E). The current elicited by 100 *μ*mol/L CACA was partially blocked by TPMPA (Fig. 2F). Collectively, these data identify a small but significant functional *ρ*‐subunit GABA_A_R response in human amygdala. Even 200 *μ*mol/L picrotoxin does not fully block 50 *μ*mol/L GABA (Fig. 2G), suggesting that the transplanted human *ρ*‐subunit GABA_A_Rs are picrotoxin insensitive. Picrotoxin block of *ρ*‐subunit GABA_A_Rs is species specific and ineffective in rat, although heterologous expression of human *ρ*‐subunit GABA_A_Rs are picrotoxin sensitive (Zhang et al. 1995).

The histogram in Figure 2D summarizes results obtained from oocytes, indicating that 50 *μ*mol/L GABA‐elicited currents were largely blocked by each of the *α*‐subunit GABA_A_R antagonists: bicuculline, SR95531 and picrotoxin (tested at the IC_max_ values). Only small percentage of the GABA currents were insensitive to these antagonists, averaging 9.8 ± 2% (*n* = 18), 7.2 ± 2% (*n* = 16), and 12 ± 4% (*n* = 18), respectively. This remaining current was very similar to the average current produced by 100 *μ*mol/L CACA, 9.3 ± 3% (*n* = 12). These results indicate that the *α*‐subunit GABA_A_R is a major inhibitory receptor in human amygdala and the *ρ*‐subunit GABA_A_R constitutes a minor component of the inhibitory input in human amygdala. With this information we could evaluate the interactions between metabotropic and the two types of ionotropic GABA receptors.

### The evidence of GABA_B_R and its inhibitory action

**Figure 3:**
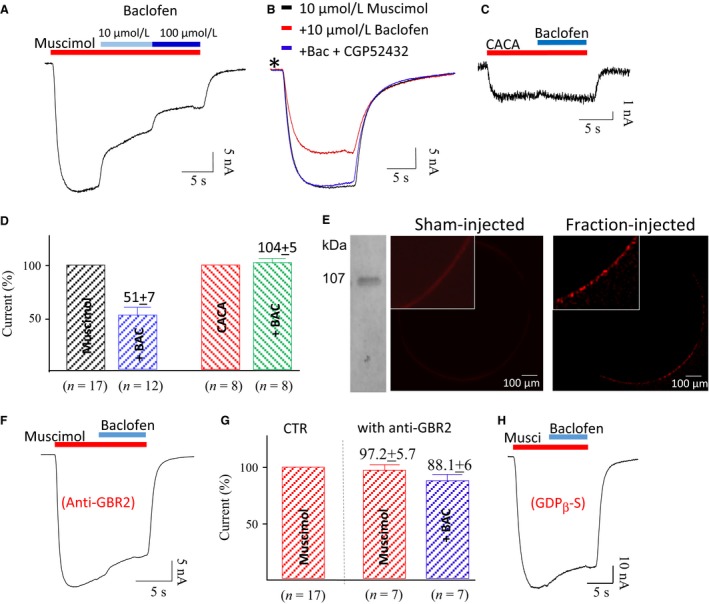
Activation of GABA_B_R inhibits *α*‐subunit GABA_A_R currents. (A) 10 *μ*mol/L and 100 *μ*mol/L baclofen inhibit muscimol (10 *μ*mol/L)‐evoked currents. (B) The suppressive effect of baclofen was blocked by 20 *μ*mol/L CGP52432, a GABA_B_R inhibitor; note that the baclofen with and without CGP52432 have no effect on the resting membrane current (see asterisk). (C) Baclofen (10 *μ*mol/L) did not suppress the CACA‐activated current. (D) Summary of the effects of 10 *μ*mol/L baclofen on the currents produced by muscimol or CACA. On average, 10 *μ*mol/L baclofen (BAC) reduced 48 ± 3% (*n* = 12) of 10 *μ*mol/L muscimol‐generated peak current, but had insignificant effects on 100 *μ*mol/L CACA‐generated currents. (E) Western blotting indicates that anti‐GBR2 antibody detected a single protein band at 107 kDa from a sample of the membrane fraction (left), and the anti‐GBR2 detection of GABA_B_Rs on the membrane surface of oocytes injected with either the membrane fraction or sham control. The inserts show magnified views of oocyte membrane. (F, G) Baclofen (10 *μ*mol/L) had small effects on the oocytes injected with the anti‐GBR2 applied membrane fractions. (H) Internal perfusion of GDP‐*β*S blocked 10 *μ*mol/L baclofen‐produced inhibition.

Both GABA_A_Rs and GABA_B_Rs are present in amygdala (Li et al. 1996) and the relative effectiveness of muscimol and GABA (see Fig. 1) suggested an interaction between the two receptor pathways. Since muscimol does not activate GABA_B_Rs, we examined the current produced by muscimol alone or in the presence of baclofen, a selective GABA_B_R agonist. Muscimol (10 *μ*mol/L) elicited currents were robustly suppressed by both 10 *μ*mol/L and 100 *μ*mol/L baclofen in a dose‐dependent manner (Fig 3A). The suppressive effect of baclofen could be fully suppressed by 20 *μ*mol/L CGP52432, a GABA_B_R antagonist (Fig. 3B). However, 10 *μ*mol/L baclofen, with or without CGP52432, produced no effect on the resting membrane currents prior to application of muscimol (Fig. 3B, asterisk), indicating that activation of GABA_B_Rs alone did not produce membrane currents in the oocytes.

The effect of baclofen on *ρ*‐subunit GABA_A_R was also tested in the transplanted oocytes. A typical example, shown in Figure 3C, indicates that 10 *μ*mol/L baclofen had no detectable effect on CACA‐elicited currents. On average, 10 *μ*mol/L baclofen suppressed 48 ± 3% (*n* = 12) of currents produced by 10 *μ*mol/L muscimol, but had no significant effect on 100 *μ*mol/L CACA‐elicited currents (*n* = 8, Fig. 3D).

To verify the membrane expression of transplanted GABA_B_Rs, immunolabeling of the oocytes was performed using the specific antibody against the residues near the C‐terminus of GBR2 (anti‐GBR2). The specificity of the antibody for human amygdala fractions was tested in Western blot assays. The anti‐GBR2 clearly recognized a single band of proteins with a molecular mass of 107 (Fig. 3E, left), consistent with the molecular mass of GABA_B_R II subunit. The anti‐GBR2 labeling results indicate that GABA_B_Rs were transported to the surface of the *Xenopus* oocytes after injecting membrane fractions from the human amgydala, but absent on the surface of oocytes injected with a sham control (Fig. 3E), demonstrating that GABA_B_Rs are not endogenously expressed in *Xenopus* oocytes.

In another approach to test the suppressive effect of baclofen on GABA_A_R currents, the anti‐GBR2 antibody was used to selectively disrupt GABA_B_R function. Functional GABA_B_Rs are heterodimers composed of GBR1 and GBR2 (Geng et al. 2013). The anti‐GBR2 antibody was applied in the membrane fraction sample with a volume ratio of 1:10,000 (antibody vs. membrane fraction sample), then injected into *Xenopus* oocytes. After 24 h, the effect of baclofen was tested on the oocytes with the standard protocol: application of 10 *μ*mol/L muscimol with and without 100 *μ*mol/L baclofen. In the presence of the anti‐GBR2, baclofen had a comparatively small effect on the muscimol‐elicited currents (Fig. 3F). On average, with anti‐GBR2 application, muscimol with and without baclofen generated about 97.2 ± 5.7% (*n* = 7) and 88.1 + 6% (*n* = 7) of the control muscimol‐elicited currents, respectively. Application of anti‐GBR2 had a minor action on muscimol‐activated GABA_A_Rs, but disrupted the actions of GABA_B_Rs (Fig. 3G).

To confirm that baclofen's effect was mediated by a G‐protein cascade, the nonhydrolyzable analog of guanosine‐5′‐diphosphate (GDP), guanosine‐5′‐O‐(2‐thiodiphosphate) trilithium salt (GDP‐*β*‐S), was injected into the oocyte. GDP‐*β*‐S (100 *μ*mol/L) injection suppressed the effect of baclofen on the GABA_A_ currents (Fig. 3H). This was also consistent with the previous report that baclofen does not act as a competitive antagonist at the GABA_A_Rs (Xi et al. 1997).

### Increasing GABA_A_ response by inhibition of GABA_B_Rs

**Figure 4:**
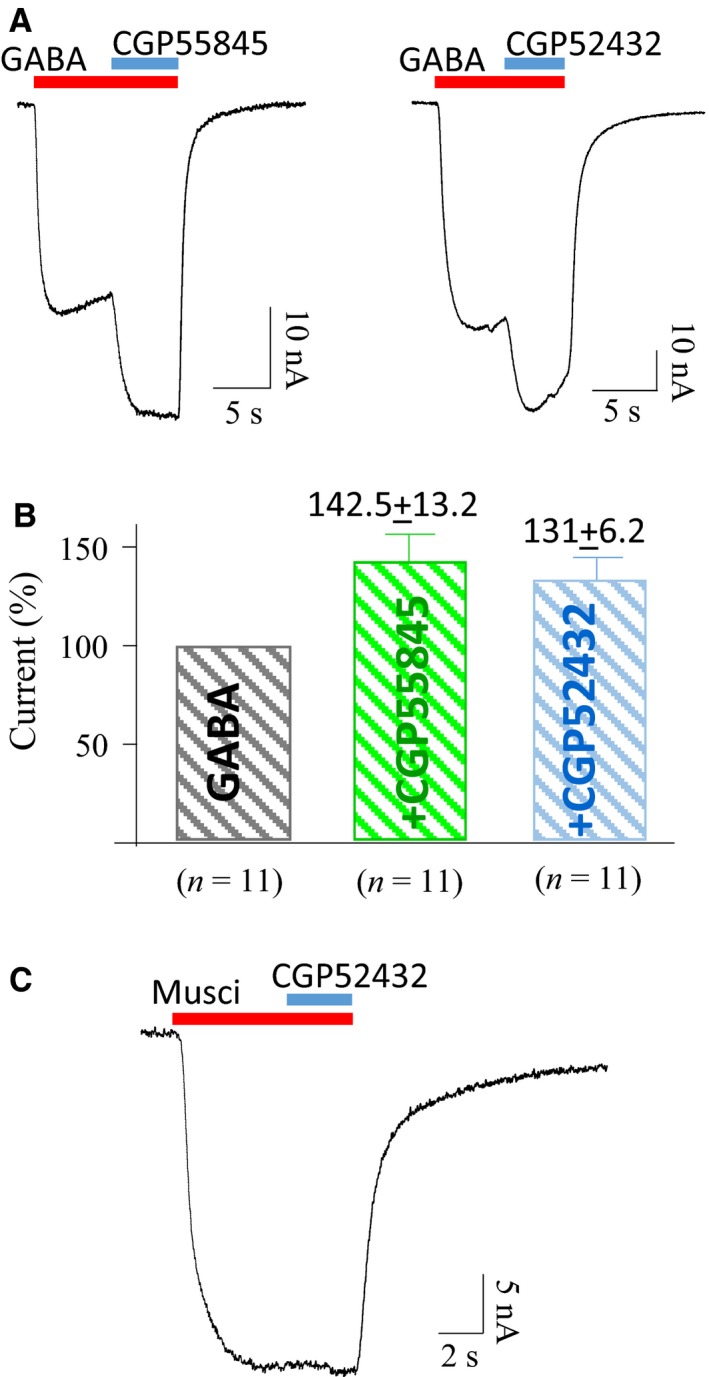
The GABA‐elicited currents were increased when blocking GABA_B_ receptors (A). Both CGP55845 and CGP52432, selective GABA_B_ antagonists, increase GABA (50 *μ*mol/L) currents in transplanted oocytes. (B) The mean percentage increase of GABA current by CGP55845 or CGP52432. In contrast, CGP52432 has no effect on 10 *μ*mol/L muscimol‐elicited currents (C).

To test the prediction that the response to GABA would be enhanced if GABA_B_Rs were not activated, the potent GABA_B_R antagonists, CGP55845 and CGP52432, were used to block GABA_B_Rs when GABA was applied. The sample recordings shown in Figure 4A indicate that 10 *μ*mol/L CGP55845 or 20 *μ*mol/L CGP52432 increased GABA (50 *μ*mol/L)‐elicited currents. This effect was present in 11 of 19 transplanted oocytes obtained from different batches. Histograms from those 11 cells show that average GABA currents in the presences of CGP55845 or CGP52432 were increased to 142.5 ± 13.2% (*n* = 11) or 131 ± 6.2% (*n* = 11), respectively (Fig. 4B). In contrast, when only GABA_A_Rs were activated with 10 *μ*mol/L muscimol, CGP52432 had no effect on muscimol‐induced currents (Fig. 4C), indicating that the GABA_B_R antagonist has no direct action on GABA_A_Rs.

### Interaction between GABA receptors in retinal ganglion cells

**Figure 5:**
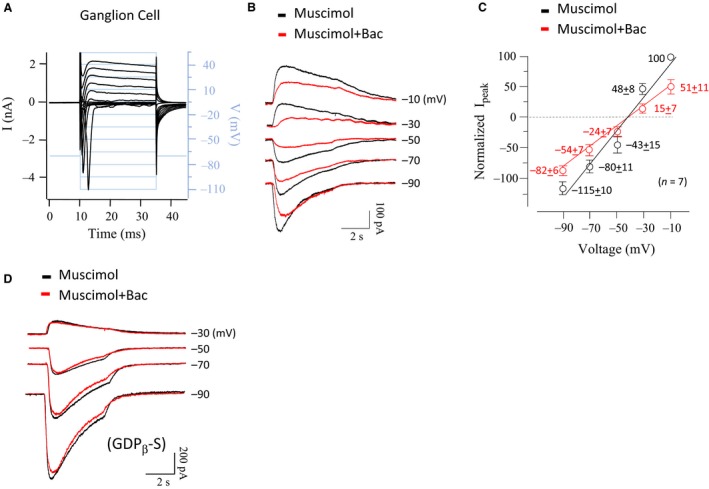
Activation of GABA_B_Rs suppressed GABA_A_R currents in mouse retinal ganglion cells. (A) Example of the current–voltage relationship of ganglion cells in whole‐cell recording. (B) Baclofen suppressed muscimol‐elicited currents in a ganglion cell clamped at various voltages. (C) Average current–voltage relationship of muscimol‐elicited currents with and without baclofen. (D) The effects of baclofen were blocked by intracellular application of GDP‐*β*‐S (100 *μ*mol/L).

The effects of GABA_B_Rs on GABA_A_R currents were also tested on neurons in the in vitro rodent retina slice preparation. Neurons were recorded in the ganglion cell layer using the whole‐cell voltage‐clamp technique and were characterized by large voltage‐activated sodium currents, typical of ganglion cells (Fig. 5A). Neurons were held at various potentials between −90 and −10 mV and either 30 *μ*mol/L muscimol or muscimol plus 10 *μ*mol/L baclofen was focally applied. A typical experiment is shown in Figure 5B, the averaged I–V curve from recordings in seven neurons is shown in Figure 5C. Baclofen suppressed muscimol‐elicited inward and outward currents (*n* = 7, Fig. 5B). When GDP‐*β*‐S (100 *μ*mol/L) was added to the pipette solution to block activation of the G‐protein cascade, the effect of baclofen became negligible (Fig 5D, *n* = 5), which is consistent with the results in Figure 3H. Because application of GDP‐*β*‐S through a recording electrode could only inhibit G‐proteins in the local cells, it is possibly that the effect of baclofen is via direct action on GABA_B_Rs in the local ganglion cells, not from network inputs.

## Discussion

### Interactions between GABA receptor subtypes

The findings, from both in vitro retina and transplanted amygdala membrane, are that the GABA_B_R can suppress the inhibitory action of GABA_A_Rs. The results extend the original observation in bullfrog dorsal root ganglion (Xi et al. 1997) to the mammalian central nervous system and indicate that this regulation may be a common feature at GABA synapses. These results contrast with findings in thalamus and dentate gyrus, where the GABA_A_R current was enhanced (Connelly et al. 2013; Tao et al. 2013). This enhancement was dependent on the presence of *δ* subunits in the GABA_A_R, a subunit that apparently is not present in amygdala (Wisden et al. 1992). It suggests that feed‐forward crosstalk between GABA receptor subtypes may be prevalent in the nervous system, but the outcome may depend on the subunit composition of the GABA_A_R.

The main mechanism of action of GABA_B_Rs in CNS is to suppress voltage‐dependent Ca^2+^ channels or to activate inward rectifying K^+^ channels. Both actions are inhibitory, reducing transmitter release or causing membrane hyperpolarization, respectively. The interesting finding here is that activation of GABA_B_Rs suppressed GABA_A_ responses and this disinhibition occurs when both receptors are present on the same cell.

The cross‐talk between receptors that we observed would have a net effect of increasing excitation. Although this seems paradoxical for an inhibitory transmitter, it has been repeatedly observed in retina. In rat retinal ganglion cells, the GABA_B_R acts to suppress an N‐type calcium channel that is linked to a BK channel. Thus, the effect of GABA_B_R stimulation is to reduce an outward potassium current, thereby promoting excitation (Garaycochea and Slaughter 2016). In salamander retina, GABA_B_Rs promote excitatory synaptic input to ganglion cells (Song and Slaughter 2010) and enhance L‐type calcium currents (Shen and Slaughter 1999). The present results provide a third GABA_B_R mechanism that can promote excitation.

The concept that GABA_B_Rs can counteract GABA_A_R inhibition is not surprising since one frequent action of metabotropic receptors is to reduce presynaptic release of GABA (Deisz and Prince 1989; Chen and van den Pol 1998; Kobayashi et al. 2012). Furthermore, the GABA_B_R activation of inward rectifying potassium channels (GIRKs) is a voltage‐dependent inhibition that diminishes with excitation. Thus, the metabotropic GABA receptor seems to have multiple mechanisms to promote excitation as well as suppress it.

### GABA receptors from human amygdala

The microtransplantation technique inserts membrane microdomains, containing an array of proteins and lipids from native tissue (Miledi et al. 2006). This not only permits examination of membrane protein function that is difficult in the native tissue, but also allows for a membrane complex to be inserted together. This is in contrast to the transfection of one or a few known genes and may be particularly relevant for GABA_B_ receptors, where a diversity of effects arise from auxiliary binding proteins (Chalifoux and Carter 2011).

Amygdala microtransplantation revealed functional expression of ionotropic receptors with the properties of *α*‐subunit and *ρ*‐subunit GABA_A_Rs. The study provides the first evidence of functional *ρ*‐subunit GABA_A_Rs in human amygdala, although the *ρ*‐subunit GABA_A_R current was only about 5% of the total GABA_A_R current. The receptors had a distinctive pharmacological profile, for example, sensitive to CACA, but only partially blocked by TPMPA. It has been reported that *ρ*‐subunit GABA_A_R‐mediated miniature IPSCs in rat amygdala are partially sensitive to TPMPA (Delaney and Sah 2001).

Microtransplanted human amygdala membrane demonstrated GABA_B_Rs based on immunohistochemistry and functional studies. Multiple reports on mammalian amygdala show GABA_B_Rs are widespread in basolateral and central amygdala (Rainnie et al. 1991; Karlsson et al. 1992; Yamada et al. 1999; Delaney and Sah 2001). The experiments indicated that the metabotropic receptor could suppress *α*‐subunit, but not *ρ*‐subunit, containing GABA_A_Rs. Since the *ρ*‐subunit GABA_A_Rs have slower kinetics than *α*‐subunit GABA_A_Rs, the GABA_B_R may alter the time‐dependent balance of GABA_A_R inhibition in the amygdala. This could be important because the two ionotropic receptors have different functions. Activation of *ρ*‐subunit GABA_A_Rs in the lateral amygdala enhances the fear learning behavior (Cunha et al. 2010), as opposed to the effect of *α*‐subunit GABA_A_R activation, which suppresses emotional learning behaviors (Liu et al. 2014). Furthermore, the GABA_A_R–GABA_B_R interaction may also depend on the subunit composition of the GABA_A_R. Baclofen enhances tonic GABA_A_R current in several brain regions where the GABA_A_R *δ*‐subunit is present (Connelly et al. 2013; Tao et al. 2013), but with the opposite effect in amygdala that lacks *δ*‐subunits (Wisden et al. 1992). Therefore, the experiments highlight the potential diversity of control mechanisms produced by crosstalk between GABAR subtypes.

## Conflict of Interest

The authors declare that they have no competing interests.
